# Potential mechanisms underlying *Enterococcus faecalis*-driven pancreatic cancer cell proliferation

**DOI:** 10.1128/mbio.03963-25

**Published:** 2026-06-11

**Authors:** Minghan Guan, Xiaoyu Guo, Mengzhen Kong, Yang Qin, Hua Xu, Xiaomin Su, Pan Wang, Xiaobing Wang

**Affiliations:** 1 National Engineering Laboratory for Resource Development of Endangered Chinese Crude Drugs in Northwest China, The Key Laboratory of Medicinal Resources and Natural Pharmaceutical Chemistry, The Ministry of Education, Shaanxi Normal University, Xi'an, China; 2 Blood Center of Shaanxi Province, Xi’an, China; Columbia University, New York, New York, USA

**Keywords:** pancreatic cancer, *E. faecalis*, cell proliferation, cell migration, EGFR

## Abstract

The development of various cancers is intricately linked with the human microbiome. Recent studies have highlighted a substantial association between pancreatic cancer and gut microbiota. However, the specific roles and underlying regulatory mechanisms of individual gut microbial species in pancreatic cancer progression remain poorly understood. *Enterococcus faecalis*, a common member of the human commensal microbiota, has been found to be enriched in the tumor tissues of pancreatic cancer patients. However, its functional contribution has not been clearly defined. In this study, we established a co-culture system involving *E. faecalis* and pancreatic cancer cells. Our results show that *E. faecalis* promoted the proliferation, migration, and invasion of pancreatic cancer cells. Following pre-treatment with *E. faecalis*, the phosphorylation level of epidermal growth factor receptor (EGFR) was markedly elevated. Inhibition of EGFR effectively suppressed the pro-proliferative effects induced by *E. faecalis*. Further investigation revealed that *E. faecalis* stimulated the production of reactive oxygen species (ROS) in pancreatic cancer cells. This ROS production might be sensed by Toll-like receptors (TLRs), leading to the activation of the EGFR signaling pathway. When cells were incubated with TLR inhibitors or ROS scavengers, both EGFR expression and its downstream pro-proliferative effects were significantly attenuated. Collectively, this study provides mechanistic insights into how *E. faecalis* contributes to pancreatic cancer progression and offers new perspectives for the development of diagnostic and therapeutic strategies targeting this microbial signaling pathway.

**IMPORTANCE:**

A diverse microbiome is closely associated with cancer, as bacterial presence has been detected in the majority of solid tumors. However, the composition, abundance, and functional profiles of the microbiota vary significantly across different tumor types, thereby exerting distinct effects on tumorigenesis and disease progression. Recent studies have shown that pancreatic cancer hosts a variety of bacterial populations, including gut-derived bacteria that may translocate to pancreatic tissue via mesenteric venous or lymphatic drainage pathways. For example, *Enterococcus* and *Enterobacter* species have been identified in the cyst fluid of patients with pancreatic cystic neoplasms. Moreover, antibodies against *Enterococcus faecalis* capsular polysaccharide have been detected in the sera of patients with pancreatitis and pancreatic cancer, and *E. faecalis* has been observed in pancreatic ducts. Despite these observations, the precise mechanisms through which *E. faecalis* influences pancreatic cancer remain unclear. Our study demonstrates that *E. faecalis* promotes pancreatic cancer cell proliferation through the activation of the Toll-like receptor-reactive oxygen species-epidermal growth factor receptor signaling pathway.

## INTRODUCTION

Pancreatic cancer is an exceptionally lethal malignancy, with rising incidence and mortality rates worldwide. Pancreatic ductal adenocarcinoma (PDAC), the most common subtype, accounts for about 90% of cases (1). Current understanding of PDAC pathogenesis remains limited, and the absence of reliable biomarkers for early detection, as well as effective preventive and therapeutic strategies, critically contributes to its poor prognosis (2).

Previous studies suggest that PDAC initiation is primarily driven by intrinsic factors such as genetic predispositions and chronic inflammation. However, accumulating evidence indicates that PDAC development and progression are also shaped by its unique tumor microenvironment (TME) (3, 4). The TME comprises a diverse array of cellular and non-cellular components, including immune cells, stromal cells, and tumor-associated microbiota (5, 6). Recent research highlights the role of the microbiome in cancer, revealing that pancreatic-associated microbes can influence PDAC onset, progression, and treatment response (7, 8). Within the TME, microbiota play complex roles: some species promote tumor growth, while others exert inhibitory effects or correlate with immunotherapy response (9–11). Understanding how different microorganisms contribute to pancreatic cancer may improve diagnosis and therapy.

The human body harbors vast and diverse microbial communities collectively referred to as the microbiome, which plays a significant role in regulating various physiological processes (12, 13). Certain microbes activate anti-tumor immunity and enhance treatment efficacy, whereas others foster an immunosuppressive TME, promoting tumor growth, metastasis, and resistance (14, 15). For example, *Fusobacterium nucleatum* correlates with poor prognosis in colorectal cancer by inducing immunosuppression (16), and *Candida albicans* influences colorectal and oral cancers via the IL-17A/IL-17RA pathway (17, 18). In pancreatic cancer, microbiome research remains early-stage, but oral, gut, and pancreatic microbial communities show potential as early biomarkers (19, 20). *Helicobacter pylori* infection increases pancreatic cancer risk, possibly through systemic inflammation and immune disruption (21, 22). *Gammaproteobacteria* produce cytidine deaminase, which reduces gemcitabine sensitivity and worsens outcomes (23). These findings indicate that microbial impact on disease depends on species identity and their interactions with the TME.

Advances in metabolomics, metagenomics, and high-throughput sequencing have clarified host-microbiota interactions (24, 25). In one observational study (26), the pancreatic microbiota of PDAC patients undergoing pancreaticoduodenectomy was analyzed. The results showed that the microbial DNA profiles in the pancreas closely resembled those in the duodenum, suggesting bacteria translocation from the intestinal tract to the pancreas via the mesentery and pancreatic duct. Analysis of pancreatic juice from 36 patients (20 with pancreatic cancer and 16 with duodenal or bile duct cancer) revealed bacterial DNA in 29 samples (27). *Enterococcus* and *Enterobacter* were mainly detected in bile, while *Enterococcus faecalis* was found in pancreatic tissues of patients with chronic pancreatitis and pancreatic cancer. Serum levels of anti-*E*. *faecalis* CPS antibodies were significantly elevated in these patients compared to healthy controls. Cultures of cyst fluid from pancreatic cancer patients showed that *E. faecalis* can invade and survive within pancreatic cells under *ex vivo* conditions (28). *E. faecalis*, a common gut opportunistic pathogen in humans and other mammals (29, 30), may contribute to pancreatitis progression and pancreatic cancer development. However, its precise roles and mechanisms require further investigation.

In this study, we analyzed publicly available tumor microbiome data sets and found significant enrichment of *E. faecalis* in pancreatic cancer tissues. To explore its functional role, we established a co-culture system of *E. faecalis* and pancreatic cancer cells to assess effects on proliferation, migration, and invasion. By integrating transcriptome profiling, cytokine arrays, bioinformatics analysis, and experimental validation, we demonstrated that the Toll-like receptor (TLR)-reactive oxygen species (ROS)-epidermal growth factor receptor (EGFR) pathway plays a crucial role in *E. faecalis-*induced cancer cell growth. These findings show how specific gut microbes promote pancreatic cancer progression and suggest therapeutic strategies targeting microbiota-driven pathways to improve outcomes.

## RESULTS

### Prevalence of *E. faecalis* in pancreatic cancer

**Fig 1:**
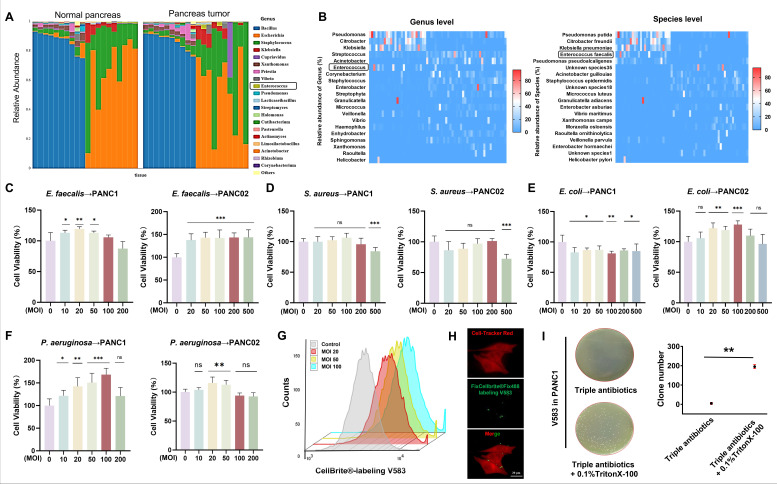
Prevalence of microorganisms in pancreatic cancer. (**A**) Comparative analysis of normal and pancreatic cancer tissues from the MicrobioTA database (PRJNA490335) revealed significant differences in microbiota composition at the genus level. (**B**) Taxonomic composition of microorganisms in PDAC tumors at the genus and species levels, with relative abundances ranked from highest to lowest among the top 20 species. (**C–F**) Viability of PANC1 and PANC02 cells 48 h after bacterial co-culture (*N* = 6). (**G**) Flow cytometry detection of Cellbrite Fix488-labeled V583 internalization in PANC1 cells across varying MOIs. (**H**) Fluorescence microscopy showing intracellular distribution of labeled V583 (green) in PANC1 cells (red). Scale bar = 20 µm. (**I**) Bacterial internalization in PANC1 cells. After 4 h of co-incubation with V583, extracellular bacteria were washed away, and cells were cultured for 24 h with antibiotics. Cells were either lysed with 0.1% Triton X-100 or left untreated, and supernatants were collected for bacterial colony-forming unit (CFU) counting (*N* = 3). *P < 0.05, **P < 0.01, ***P < 0.001.

We conducted a comparative analysis of 20 normal and 20 pancreatic cancer tissue samples from the MicrobioTA database (PRJNA490335), revealing significant differences in microbial composition between tumors and normal controls (Fig. 1A). Several bacterial genera, including *Enterococcus*, were more abundant in tumor tissues. Further analysis of data from 65 PDAC samples (23) showed distinct alterations in microbial composition at both genus and species levels (Fig. 1B). Specifically, *Pseudomonas putida*, *Klebsiella pneumoniae*, and *E. faecalis* were consistently enriched in PDAC tissues. To evaluate the functional implications of these enriched taxa, we selected representative strains based on abundance and clinical relevance, including Gram-positive species (*E. faecalis* V583, *Staphylococcus aureus*) and Gram-negative species (*Escherichia coli*, *Pseudomonas aeruginosa*). *In vitro*, we established a co-culture system in which each strain was incubated with pancreatic cancer cells for 3–4 h. Extracellular bacteria were subsequently eliminated using a triple antibiotic treatment. Following 48 h of post-infection culture, cell viability was assessed using the MTT assay. Our results showed that *E. faecalis* V583 significantly increased the viability of both human (PANC1) and murine (PANC02) pancreatic cancer cell lines at specific multiplicities of infection (MOIs) (Fig. 1C). In contrast, *Staphylococcus aureus* had little effect on cell viability, except for inducing cytotoxicity at high MOIs (Fig. 1D). *E. coli* slightly inhibited the growth of PANC1 cells but promoted the proliferation of PANC02 cells (Fig. 1E). *Pseudomonas aeruginosa* significantly enhanced PANC1 viability, although its effect on PANC02 cells was less pronounced (Fig. 1F). Thus, although *E. faecalis* was not universally dominant, its repeated detection and consistent association with PDAC across multiple data sets suggest a potential functional role in shaping the PDAC microenvironment, meriting further mechanistic investigation.

To examine the interaction between *E. faecalis* and pancreatic cancer cells, we fluorescently labeled the V583 strain with Cellbrite Fix488 and co-cultured it with PANC1 cells at varying MOIs for 4 h. Uninfected PANC1 cells served as controls. Following removal of extracellular bacteria by washing, flow cytometry was used to quantify internalized bacteria. Results showed that green fluorescent-labeled V583 successfully entered PANC1 cells (Fig. 1G). Fluorescence microscopy confirmed the presence of green-labeled bacteria within PANC1 cells pre-stained with Cell Tracker Red (Fig. 1H). Moreover, cell lysis with Triton X-100 followed by bacterial culture yielded more colonies than the non-lysed group (Fig. 1I), confirming bacterial internalization. Together, these results indicate that under short-term co-culture conditions, *E. faecalis* V583 can invade and reside within pancreatic cancer cells. Given that extracellular bacteria were eliminated by triple antibiotic treatment, the increased cell viability at 48 h is likely due to intracellular colonization.

### *E. faecalis* promoted the proliferation, migration, and invasion of pancreatic cancer cells

**Fig 2:**
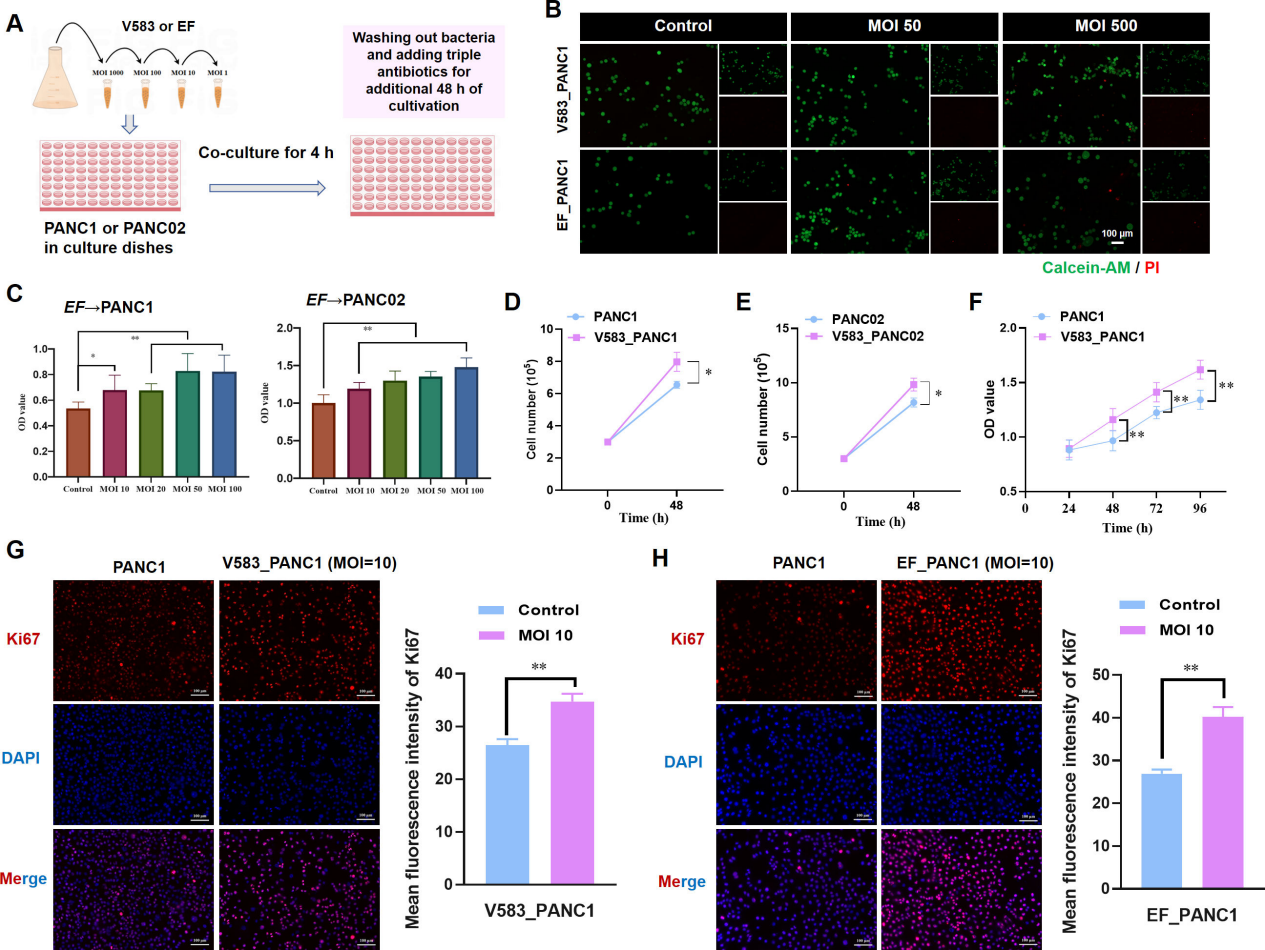
*E. faecalis* promoted the proliferation of pancreatic cancer cells. (**A**) An *in vitro* co-culture system was established to explore the influence of *E. faecalis* on pancreatic cancer cells. PANC1 and PANC02 cells were briefly co-cultured with V583 or EF at various MOIs, followed by removal of extracellular bacteria via washing and further culture for 48 h with a triple-antibiotic cocktail. (**B**) Cell survival was assessed by Calcein-AM/PI staining after 4 h of co-culture. Scale bar = 100 µm. (**C**) The impact of *E. faecalis* on the viability of pancreatic cancer cells was assessed using MTT assay at 48 h. (**D and E**) PANC1 or PANC02 cell numbers were quantified using a cell counter after 48 h with or without V583 stimulation. (**F**) The proliferation of PANC1 cells was assessed by EdU assay at multiple time points post-V583 infection. (**G and H**) Ki67 immunofluorescence staining and quantitative analysis were performed 48 h after infection with V583 (**G**) or EF (**H**) to assess PANC1 proliferation. *P < 0.05, **P < 0.01 (*N* = 6).

To investigate the impact of *E. faecalis* on pancreatic cancer cells, we established a short-term co-culture system based on relevant literature in tumor microbiology (31, 32) and utilized triple-antibiotic treatment to eliminate extracellular bacteria. Clinical strains EF and V583 were co-cultured with PANC1 and PANC02 cells (Fig. 2A). Cell survival was assessed after 4 h of co-culture using calcein-AM/PI staining, which revealed no significant effect on PANC1 viability at MOI ≤ 500 (Fig. 2B). MTT assays demonstrated that EF enhanced metabolic activity in both PANC1 and PANC02 cells in a dose-dependent manner within the MOI range of 10–100 (Fig. 2C). Cell counting revealed that at an MOI of 10, V583 significantly increased the number of colonies formed by both cell lines (Fig. 2D and E). EdU assay indicated that PANC1 cell proliferation was significantly enhanced within 96 h post-infection with V583 (Fig. 2F). Furthermore, Ki67 immunofluorescence staining confirmed that both V583 and EF increased the proportion of Ki67-positive cells (Fig. 2G and H), suggesting an overall enhancement in cell proliferative capacity. Collectively, these results demonstrate that the selected *E. faecalis* strains can significantly promote the long-term proliferation of pancreatic cancer cells following brief co-culture exposure.

**Fig 3:**
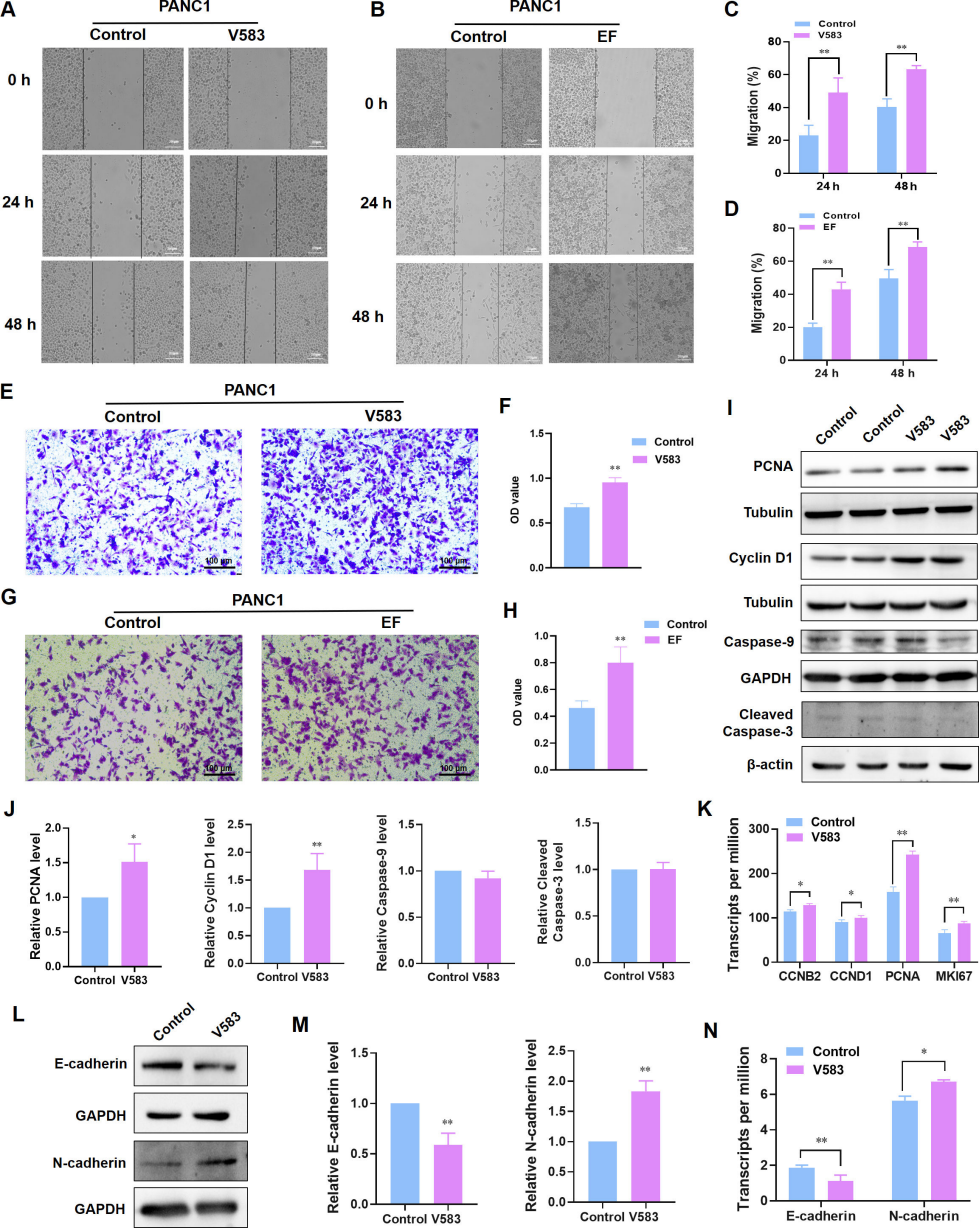
*E. faecalis* enhanced the migration and invasion of pancreatic cancer cells. (**A**) Wound healing assay showing PANC1 cell migration after V583 infection (scale bar = 200 µm). (**B**) Wound healing assay assessing the effect of EF infection on PANC1 cell migration (scale bar = 200 µm). (**C, D**) Quantification of wound closure in panels **A** and **B**. (**E**) Transwell assay evaluating the invasive potential of PANC1 cells after V583 infection (scale bar = 100 µm). (**F**) Statistical analysis of panel **E**. (**G**) Transwell assay assessing invasion of EF-infected PANC1 cells (scale bar = 100 µm). (**H**) Statistical analysis of panel **G**. (**I**) Western blot analysis of proliferation markers (PCNA, cyclin D1) and apoptosis markers (caspase-9, cleaved caspase-3) in PANC1 cells following V583 stimulation. (**J**) Quantitative analysis of panel **I**. (**K**) RNA-seq data showing upregulation of proliferation-related genes (CCNB2, CCND1, PCNA, MKI67) in PANC1 cells 48 h post-V583 treatment. (**L**) Western blot analysis of E-cadherin and N-cadherin expression in V583-treated PANC1 cells. (**M**) Quantitation of panel **L**. (**N**) RNA-seq results showing altered expression of E-cadherin and N-cadherin genes in PANC1 cells after V583 stimulation. *P < 0.05, **P < 0.01 (*N* = 4).

To investigate the effects of *E. faecalis* on the functional behaviors of pancreatic cancer cells, we performed a series of assays. Wound healing assays showed a significant enhancement in the migratory capacity of pancreatic cancer cells following *E. faecalis* infection (Fig. 3A through D). Specifically, at 24 and 48 h post-infection, the migration rates in the V583-treated group were 49.03% ± 9.02% and 63.25% ± 2.25%, respectively, which were markedly higher than those in the control group (23.00% ± 6.20% and 40.33% ± 5.01%) (Fig. 3A and C). Similarly, EF-treated PANC1 cells also exhibited significantly increased migration compared with the untreated group (Fig. 3B and D). To assess the invasive potential of PANC1 cells after exposure to V583 or EF, Transwell assays were conducted using Matrigel-coated chambers. The results showed that the OD value of the V583-treated group at 48 h was 0.95 ± 0.05, compared to 0.68 ± 0.04 in the control group (Fig. 3E and F). For the EF-treated group, the OD value at 48 h was 0.80 ± 0.12 versus 0.46 ± 0.05 in the control group (Fig. 3G and H). These findings indicate that both V583 and EF significantly promoted the invasion of pancreatic cancer cells. Western blot analysis revealed upregulation of proliferation markers PCNA and cyclin D1 (Fig. 3I and J). RNA-seq data showed that proliferation-related genes, such as CCNB2, CCND1, PCNA, and MKI67, were significantly upregulated upon *E. faecalis* infection (Fig. 3K), supporting enhanced proliferative capacity. No significant changes were observed in the expression levels of apoptosis-related proteins caspase-9 and cleaved caspase-3. However, expression of migration- and invasion-related proteins E-cadherin and N-cadherin was significantly altered (Fig. 3L and M), consistent with transcriptomic profiles (Fig. 3N). These results demonstrate that *E. faecalis* promotes proliferation, migration, and invasion of pancreatic cancer cells.

### Signaling pathways involved in *E. faecalis*-promoted pancreatic cancer cell growth

**Fig 4:**
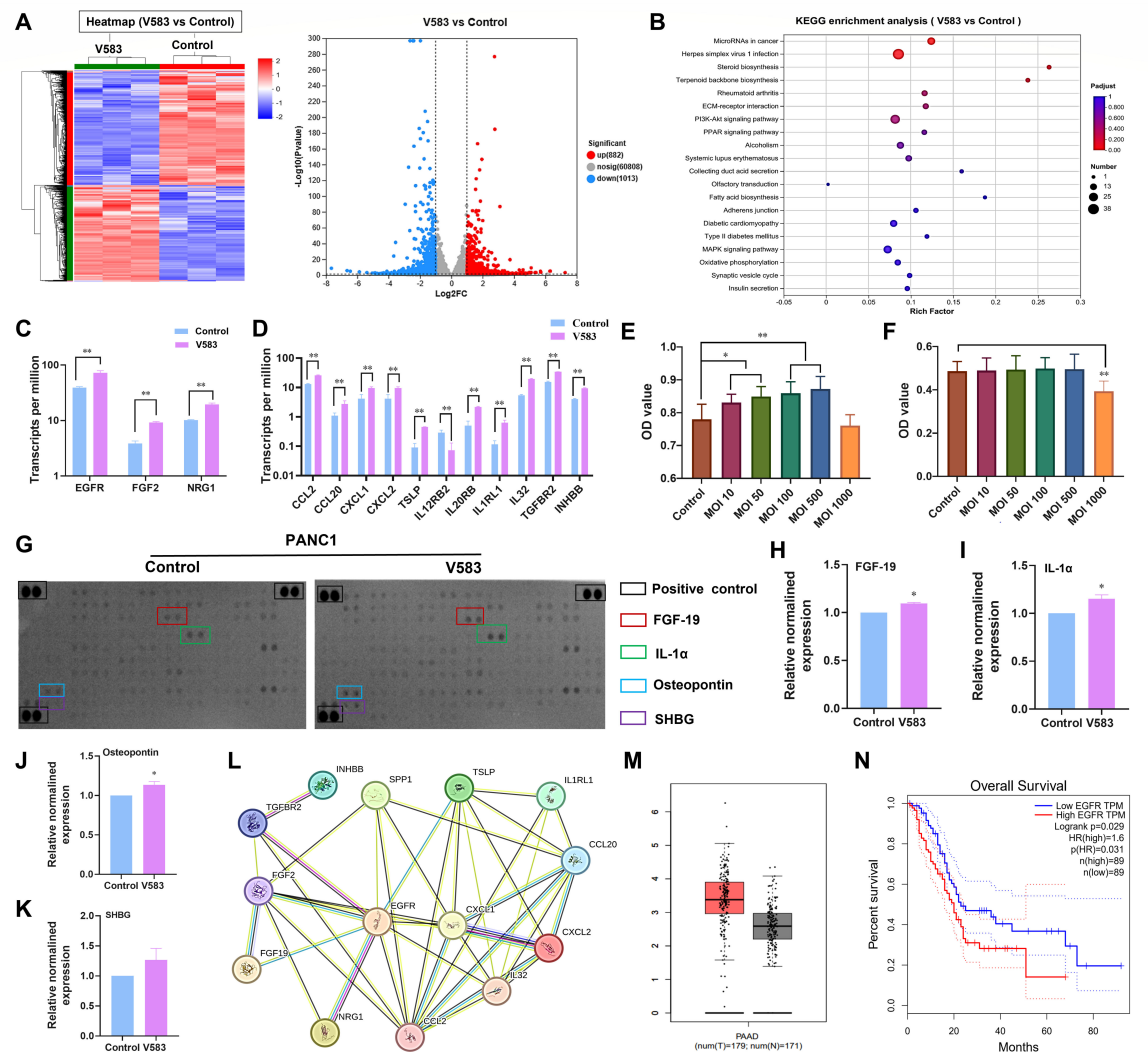
Signaling pathways involved in *E. faecalis* V583-promoted PANC1 cell growth. (**A**) Clustering heatmap and volcano plot of RNA-seq data. (**B**) KEGG pathway enrichment analysis. The *y*-axis lists enriched pathways, and the *x*-axis shows the ratio of differentially expressed genes to total annotated genes. Larger values indicate greater enrichment. Dot size reflects the number of genes involved, and color represents corrected *P* values. (**C**) Expression of key genes in cell growth-related signaling pathways in control vs V583-treated PANC1 cells (*N* = 3). (**D**) Expression of genes in the cytokine-cytokine receptor interaction pathway (*N* = 3). (**E**) MTT assay assessing viability of PANC1 cells cultured with conditioned medium from V583-infected PANC1 cells (*N* = 6). (**F**) MTT assay evaluating viability after exposure to conditioned medium from V583 alone (*N* = 6). (**G**) Solid-phase cytokine microarray of conditioned medium from PANC1 cells with or without V583 co-culture. Black box: positive control; red box: FGF-19; green box: IL-1α; blue box: SPP1; purple box: SHBG. (**H–K**) Quantification of FGF-19, IL-1α, SPP1, and SHBG levels in conditioned medium. (**L**) STRING-based interaction network of key molecules of interest. (**M**) GEPIA analysis of EGFR expression in pancreatic cancer vs normal tissues. (**N**) Association between high EGFR expression and reduced overall survival in pancreatic cancer patients. *P < 0.05, **P < 0.01.

To explore transcriptomic changes in PANC1 cells induced by V583 stimulation, RNA-seq was conducted. At 48 h post-stimulation, 695 genes were upregulated, and 828 genes were downregulated (Fig. 4A). KEGG enrichment analysis revealed significant alterations in pathways linked to pancreatic cancer progression, including steroid biosynthesis, fatty acid biosynthesis, extracellular matrix (ECM)-receptor interaction, PI3K-Akt, and MAPK signaling (Fig. 4B). Expression of receptor signaling genes associated with proliferation like EGFR, FGF2, and NRG1 was significantly increased (Fig. 4C). In addition, several cytokines showed altered expression patterns in PANC1 cells co-cultured with V583. Specifically, within the cytokine-cytokine receptor interaction pathway, the expression levels of CCL2, CCL20, CXCL1, CXCL2, TSLP, IL32, IL20RB, IL1RL1, TGFBR2, and INHBB were significantly increased, with TSLP exhibiting the most pronounced upregulation (Fig. 4D). To evaluate the functional impact of secreted factors, PANC1 cells were cultured with conditioned medium collected 4 h after co-incubation with V583. MTT assay at 48 h showed this conditioned medium significantly promoted cell proliferation (Fig. 4E), whereas the medium derived from V583 alone had no such effect (Fig. 4F). The findings suggest that the secretion of specific cytokines during short-term co-cultivation may contribute to PANC1 cell proliferation. Further cytokine array analysis of the supernatant from V583-co-cultured PANC1 cells revealed that V583 infection specifically enhanced the secretion of FGF-19, IL-1α, and osteopontin (SPP1) (Fig. 4G through K). All three molecules have been implicated in pancreatic cancer progression. Notably, IL-1α can promote tumor growth by modulating TSLP expression. Protein-protein interaction analysis using the STRING database indicated that EGFR interacts with multiple relevant molecules (Fig. 4L). Subsequently, GEPIA database analysis of 179 tumor and 171 normal samples confirmed higher EGFR expression in pancreatic cancer tissues (Fig. 4M) and its association with poorer overall survival (Fig. 4N).

### EGFR contributed to *E. faecalis*-mediated regulation of pancreatic cancer cell growth

**Fig 5:**
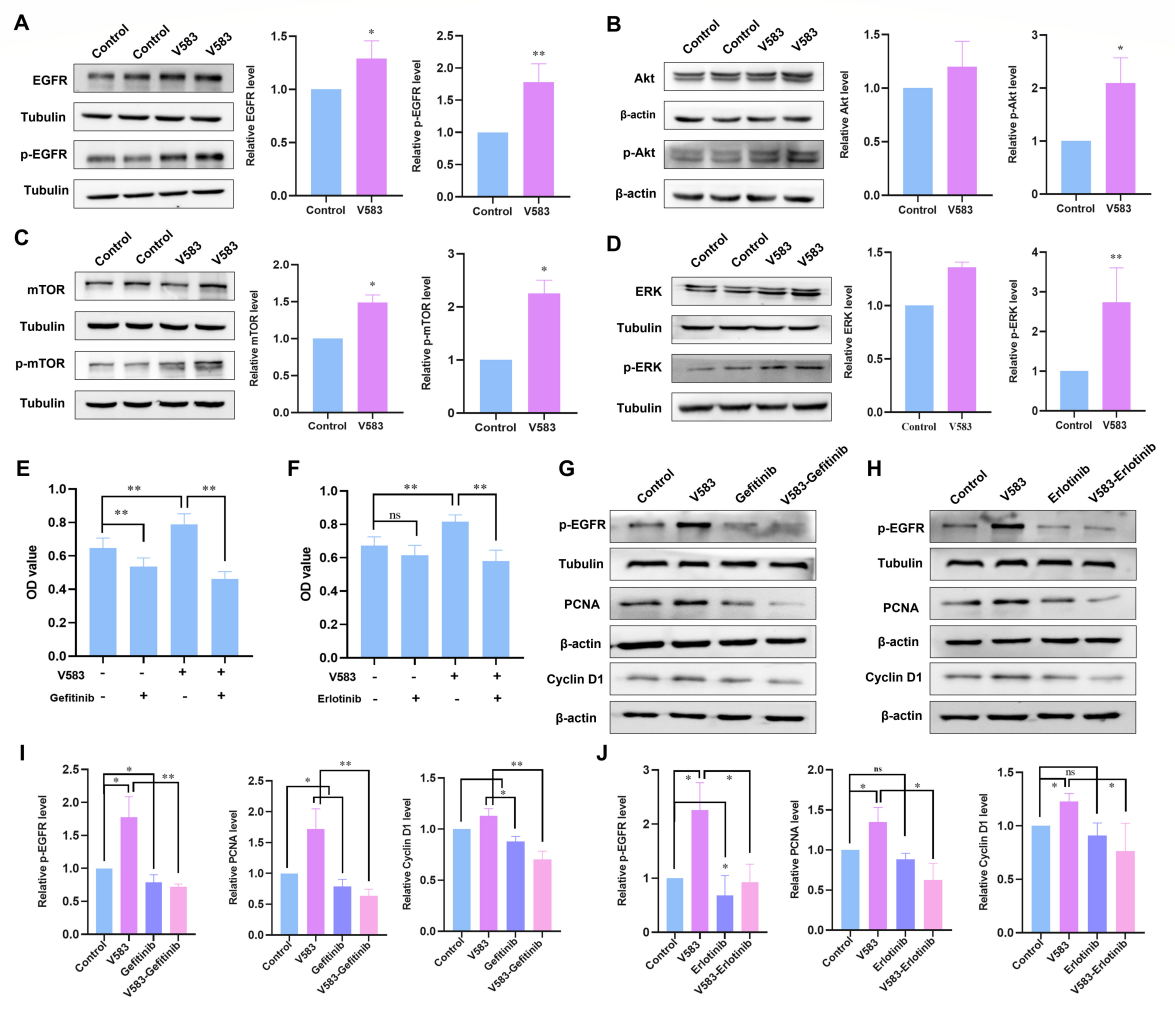
Role of EGFR in *E. faecalis*-mediated promotion of pancreatic cancer cell growth. (**A**) Western blot analysis of EGFR and p-EGFR expression in PANC1 cells after V583 infection, with quantitative data. (**B**) Impact of V583 infection on Akt and p-Akt expression in PANC1 cells, with quantitative analysis. (**C**) mTOR and p-mTOR expression in PANC1 cells after V583 infection, with quantitative assessment. (**D**) ERK and p-ERK expression in PANC1 cells post-V583 infection, supported by quantification. (**E**) MTT assay showing the effect of V583 infection on PANC1 cell viability in the presence or absence of the EGFR inhibitor gefitinib. (**F**) MTT assay showing the effect of V583 infection on PANC1 cell viability with or without the EGFR inhibitor erlotinib. (**G**) Effects of V583 on p-EGFR, PCNA, and cyclin D1 expression in PANC1 cells following treatment with gefitinib. (**H**) Effects of V583 on the expression levels of p-EGFR, PCNA, and cyclin D1 in PANC1 cells following treatment with erlotinib. (**I and J**) Quantitative analyses of panels **G** and **H**, respectively. *P < 0.05, **P < 0.01 (*N* = 3).

EGFR serves as a critical therapeutic target in clinical oncology, modulating multiple signaling cascades, such as PI3K/Akt and MAPKs, which govern essential cellular processes including growth, proliferation, and motility (33). To investigate the role of EGFR in *E. faecalis*-mediated regulation of pancreatic cancer cell growth, we assessed alterations in EGFR expression and its downstream signaling molecules in PANC1 cells co-cultured with V583. Our results demonstrated that at 48 h post-coculture, both total EGFR and phosphorylated EGFR (p-EGFR) were significantly elevated (Fig. 5A). Moreover, the levels of downstream signaling proteins, including p-Akt, p-mTOR, mTOR, and p-ERK, were also markedly increased (Fig. 5B through D). To further evaluate the involvement of the EGFR signaling pathway, we employed two specific EGFR inhibitors, gefitinib and erlotinib. MTT assays indicated that the proliferative effect induced by V583 on PANC1 cells was significantly attenuated in the presence of these inhibitors (Fig. 5E and F). Specifically, V583 enhanced the expression of p-EGFR, PCNA, and cyclin D1 compared with the control group; however, this enhancement was effectively reversed by treatment with gefitinib or erlotinib (Fig. 5G through J). These findings suggest that EGFR activation is a central mechanism through which *E. faecalis* influences the growth of pancreatic cancer cells.

**Fig 6:**
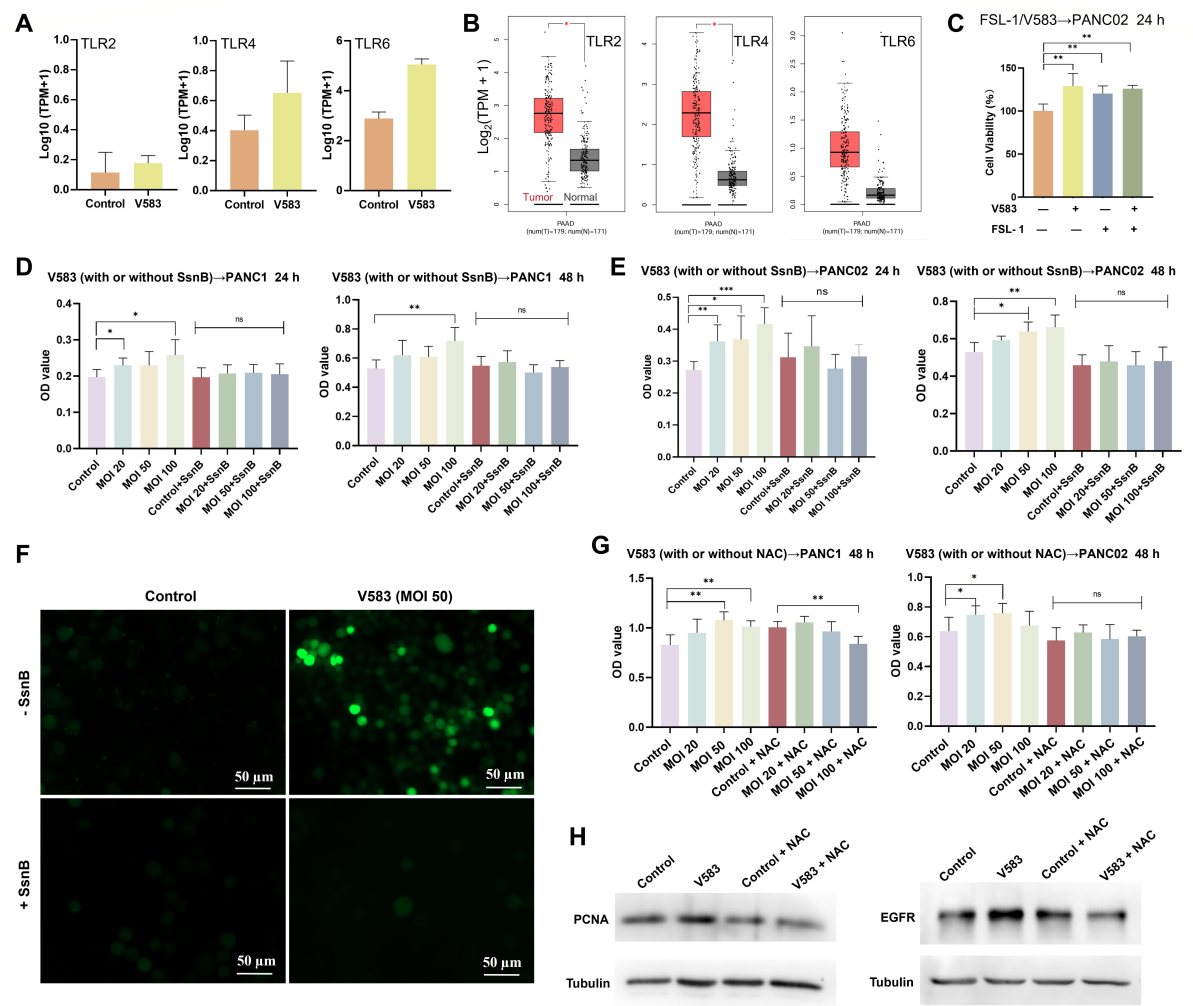
*E. faecalis* regulated pancreatic cancer cell growth via the TLR-ROS-EGFR pathway. (**A**) TLRs gene expression changes in PANC1 cells after V583 infection (*N* = 3). (**B**) TLR expression in normal vs pancreatic cancer tissues using GEPIA data. (**C**) PANC02 cell viability at 24 h after treatment with V583, FSL-1 (0.05 μM), or both (V583 pre-treatment followed by FSL-1). (**D and E**) V583 effects on PANC1 and PANC02 cell viability with or without SsnB, a TLR2/4 inhibitor (*N* = 6). (**F**) SsnB reduces V583-induced ROS accumulation in PANC1 cells. (**G**) Effect of NAC on V583-induced cell viability (*N* = 6). (**H**) EGFR and PCNA expression in V583-infected PANC1 cells after ROS scavenging. *P < 0.05, **P < 0.01, ***P < 0.001.

Toll-like receptors (TLRs) are essential pattern recognition receptors that recognize pathogen-associated molecular patterns (PAMPs) and play a central role in host defense. Recent studies show that TLR signaling bridges microbial interactions with tumor cells, linking innate immunity to inflammation-driven tumorigenesis (34, 35). To explore how *E. faecalis* promoted pancreatic cancer cell growth through EGFR activation, we analyzed RNA-Seq data and found that TLR2, TLR4, and TLR6 were upregulated following V583 infection (Fig. 6A), implicating TLR signaling in EGFR regulation. Analysis of the GEPIA database data further revealed elevated expression of TLR2, TLR4, and TLR6 in pancreatic cancer tissues compared with normal tissues (Fig. 6B), suggesting their involvement in malignant progression. FSL-1, a specific agonist of TLR2/6, is known to activate NF-κB signaling and induce inflammatory and proliferative responses. Our results (Fig. 6C) show that FSL-1 alone induced modest pancreatic cancer cell proliferation, likely due to its indirect mechanism involving inflammatory mediator release rather than direct cell cycle stimulation. Notably, when cells were pre-exposed to *E. faecalis* V583 prior to FSL-1 treatment, no significant additive proliferative effect was observed. This may be due to competition between V583 components (e.g., lipoteichoic acid) and FSL-1 for TLR binding, suggesting that *E. faecalis* activates TLR pathways in a way that overlaps functionally with FSL-1 but differs mechanistically. Previous studies indicate that TLR signaling enhances EGFR expression and activity by modulating intracellular ROS levels (36), which are critical regulators of cancer cell behavior in the tumor microenvironment. To validate the TLR-ROS-EGFR signaling axis, we performed inhibitory experiments. Pharmacological inhibition of TLR2/4 with Sparstolonin B (SsnB) attenuated *E. faecalis*-induced pro-proliferation of pancreatic cancer cells (Fig. 6D and E). DCFH-DA staining demonstrated increased ROS production in PANC1 cells upon V583 infection (Fig. 6F), an effect that was reversed by SsnB, indicating TLR-dependent ROS generation. Furthermore, treatment with N-acetylcysteine (NAC), a potent ROS scavenger, significantly suppressed V583-induced cell proliferation (Fig. 6G) and reduced expression of EGFR and PCNA (Fig. 6H). These findings suggest that ROS may serve as a key intermediary in the TLR–EGFR signaling cascade, through which *E. faecalis* drives pancreatic cancer cell progression.

### *E. faecalis* promoted pancreatic tumor growth *in vivo*

**Fig 7:**
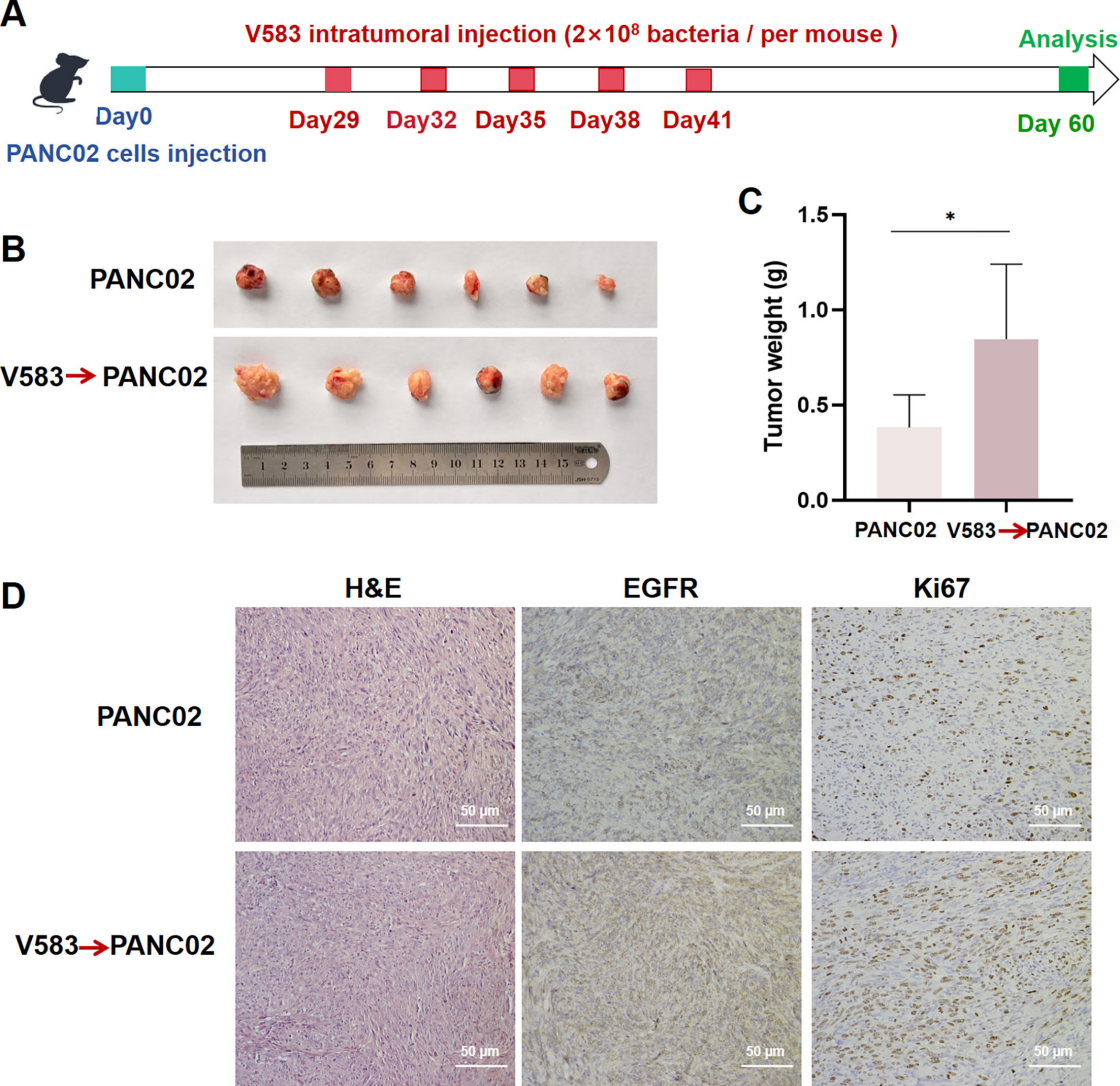
The impact of *E. faecalis* on pancreatic tumor growth *in vivo*. (**A**) Schematic of the *in vivo* experimental model for V583 infection in pancreatic cancer. (**B**) Representative *ex vivo* tumor images at day 60, showing visible differences between V583 and control groups. (**C**) Comparison of tumor weights between the V583-inoculated and control groups at day 60. *P < 0.05 (*N* = 6). (**D**) H&E staining and immunohistochemical analysis of EGFR and Ki67 expression in tumor tissues.

To evaluate the proliferative effects of *E. faecalis* on pancreatic cancer cells *in vivo*, PANC02 cells were subcutaneously implanted into C57BL/6 mice. When the tumor volume reached approximately 100 mm³, multiple intratumoral injections of V583 were administered (Fig. 7A). Tumor samples were collected and analyzed on day 60. The results showed that V583 promoted pancreatic cancer tissue growth *in vivo* (Fig. 7B and C). The tumor weight in the V583-treated group was markedly higher than that in the control group. Histological analysis by H&E staining revealed a more compact cellular arrangement in the tumor tissues of V583-injected mice. Furthermore, immunohistochemical staining demonstrated a significant upregulation of Ki67 and EGFR expression in V583-infected tumors (Fig. 7D), indicating enhanced cell proliferation. These *in vivo* findings are consistent with our *in vitro* observations, further supporting the role of *E. faecalis* in promoting pancreatic cancer cell proliferation.

## DISCUSSION

The human microbiome has emerged as a critical focus in cancer research, with growing evidence indicating its involvement in tumor initiation, progression, and therapy response. Each tumor type harbors a distinct microbial profile (37, 38). Pancreatic cancer tissues exhibit marked microbial dysbiosis compared to normal tissues, characterized by significant alterations in the diversity and composition of bacterial and fungal communities (39). This intratumoral microbiome has been closely linked to tumor progression, treatment efficacy, and prognosis (40). Studies have shown that the abundance of *Enterococcus* is significantly elevated in patients with chronic pancreatitis or pancreatic cancer compared with healthy controls, with *E. faecalis* being enriched (27, 41). As a common Gram-positive bacterium found in the oral cavity and gut, *E. faecalis* has been linked to colorectal, liver, and oral cancers (42–44). It produces virulence factors and ROS, causing genomic instability and mutations that drive tumorigenesis. Its gelatinase can disrupt the epithelial barrier, promoting chronic inflammation (45). As gut microbes can reach the pancreas *via* routes, such as ductal reflux, investigating *E. faecalis* in pancreatic cancer is essential.

In this study, we utilized a co-culture model to assess the effects of two clinical *E. faecalis* strains on pancreatic cancer cells. Our results showed that *E. faecalis* significantly promoted cancer cell proliferation, migration, and invasion. RNA-seq and KEGG analysis revealed involvement of the ECM-receptor interaction, PI3K-Akt, and MAPK pathways in V583-induced cell growth. ECM components enhance cell adhesion, migration, and epithelial-mesenchymal transition (EMT) through integrin signaling and contribute to chemoresistance (46). The PI3K/Akt and MAPK pathways regulate proliferation, metabolism, motility, and stress responses (47). We found that *E. faecalis* upregulated PCNA and cyclin D1 and activated the EGFR/PI3K/Akt/ERK cascade, indicating it accelerates tumor progression. Studies have shown that microbial agents can drive tumor progression via PI3K-Akt activation. For example, *Fusobacterium nucleatum* promotes colorectal carcinogenesis through RadD binding with CD147 and subsequent PI3K-Akt cascade (48). It also enhances the occurrence of esophageal cancer by upregulating IL-32/PRTN3, which activates the same pathway (49). Notably, V583 infection increased the expression of EGFR. As a key tyrosine kinase receptor, EGFR triggers pro-proliferative signals and enhances cell survival. Studies have shown that *Helicobacter pylori* and *Mycoplasma hyorhinis* infections can activate EGFR during carcinogenesis (50, 51), supporting a link between bacterial infection and EGFR activation in tumors. In pancreatic cancer, elevated EGFR correlates with poor survival. Aberrant EGFR activation can induce Kras mutations, leading to Ras-MAPK and PI3K-Akt signaling, disrupting pancreatic acinar cell homeostasis and promoting duct-like cell transformation, thereby contributing to PDAC development (52, 53). Therefore, elucidating the potential involvement of EGFR in *E. faecalis*-induced pancreatic cancer cell growth is essential for understanding the underlying mechanisms of microbe-associated tumor progression.

In V583-infected PANC1 cells, cytokines, including CCL2, CCL20, CXCL1, CXCL2, TSLP, and IL-32, were upregulated. TSLP showed the most pronounced increase and was closely linked to EGFR activation. Culturing PANC1 cells in conditioned medium from infected cells sustained proliferation, suggesting that *E. faecalis* promotes tumor growth by inducing tumor cell secretion of specific cytokines. Microarray analysis revealed that co-culture with V583 increased secretion of FGF-19, IL-1α, and SPP1, all of which are associated with EGFR signaling. Treatment with EGFR inhibitors, such as gefitinib or erlotinib, significantly attenuated V583-induced cell proliferation. The expression levels of phosphorylated EGFR, PCNA, and cyclin D1 were also reduced, reinforcing the essential role of EGFR in mediating *E. faecalis*-driven tumor promotion.

In tumor microbiology, growing evidence shows that intra-tumoral bacteria are not passive bystanders but actively contribute to tumor progression through a “recognition-signal amplification-effect activation” cascade (54, 55). The cell wall components of *E. faecalis*, such as lipoteichoic acid, act as classical PAMPs (56, 57) and are recognized by host TLR2, often in complex with TLR6, enhancing signal transduction. Moreover, *E. faecalis* infection causes tumor cell damage, resulting in the release of damage-associated molecular patterns, which activate TLR4 or TLR8 and establish an “indirect activation” mechanism. Activation of TLRs initiates downstream signaling and leads to the production of inflammatory cytokines and ROS (58). The biological effects of ROS are highly concentration-dependent (59). While high levels of ROS typically induce apoptosis, those induced by *E. faecalis* in the oxidative stress-rich pancreatic tumor microenvironment remain moderately elevated. These ROS levels do not trigger cell death but instead function as secondary messengers to activate EGFR, which in turn stimulates the PI3K-AKT and MAPK pathways. Inhibitor experiments demonstrated that blocking TLR2/4 significantly reduced V583-induced cell proliferation and ROS accumulation, underscoring the essential involvement of TLR signaling. Furthermore, NAC-mediated ROS scavenging suppressed both V583-induced viability increase and EGFR activation, confirming the intermediary role of ROS in this process.

In summary, *E. faecalis* is a common opportunistic pathogen implicated in the development of multiple cancers. Using a co-culture system, we demonstrated that V583 enhanced pancreatic cancer cell malignancy. Mechanistically, *E. faecalis* activates the TLR-ROS-EGFR axis, triggering downstream pro-proliferative pathways such as PI3K-Akt and MAPK, and upregulating key regulators like cyclin D1. *In vivo,* V583 promoted pancreatic tumor growth and EGFR expression. Additionally, V583 stimulated tumor cells to secrete a range of cytokines that sustain its pro-proliferative effects. These findings provide valuable insights into how *E. faecalis* influences pancreatic cancer progression and highlight potential therapeutic strategies targeting microbial components and their associated signaling pathways.

## MATERIALS AND METHODS

### Cell lines and bacterial strains

The human pancreatic cancer cell line PANC-1 and the murine cell line PANC02 were cultured in DMEM high-glucose medium supplemented with 10% fetal bovine serum, 1% L-glutamine, and 1% penicillin-streptomycin. All cultures were maintained at 37°C with 5% CO_2_. *Enterococcus faecalis* V583 (ATCC 700802) was provided by the Chunhua Qiushi research group at Nanchang University. *E. faecalis* EF (ATCC 19433) was obtained from BeNa Culture Collection (China). *Staphylococcus aureus* (CMCC 26003) was supplied by the Shaanxi Institute of Microbiology. These strains were cultured in brain heart infusion (BHI) medium. *Escherichia coli* (NISSLE 1917) and *Pseudomonas aeruginosa* (BNCC 125486) were obtained from BNCC (China) and cultured in Luria-Bertani (LB) medium. All strains were used for experiments during their logarithmic growth phase.

### Co-culture of *E. faecalis* and pancreatic cancer cells

PANC1 or PANC02 cells were seeded into culture plates and allowed to adhere overnight. The following day, the supernatant was removed, and serum-free medium containing either V583 or EF at various MOIs was added for co-incubation for approximately 4 h. Afterward, extracellular bacteria were washed with PBS, and the cells were cultured in DMEM complete medium supplemented with a triple antibiotic cocktail (1% penicillin-streptomycin and 200 μg/mL gentamicin) under standard conditions for 48 h before experimental assays were conducted. Additional experiments were also carried out at 24 and 72 h post-co-culture.

To assess bacterial colonization within pancreatic cancer cells, V583 bacteria were labeled with Cellbrite Fix488 (Biotium, USA), and PANC-1 cells were stained with 5 μM Cell Track Red (MKBio Co., Shanghai, China). V583 and PANC-1 cells were co-incubated at an MOI of 100 for 4 h. After co-incubation, the cells were washed with PBS to remove unbound bacteria, fixed with paraformaldehyde, and examined under an inverted fluorescence microscope. In parallel experiments, V583 was labeled with Cellbrite Fix488 and co-incubated with PANC-1 cells at varying MOIs for 4 h. Intracellular fluorescence intensity was then measured using a flow cytometer (cytoFLEX S, Beckman).

### Publicly accessible databases

The MicrobioTA database is a comprehensive platform for analyzing tumor-associated microbiomes (60). It enables comparative analysis of microbial genomic sequences from diverse human and murine tissue samples and supports basic investigations into bacterial abundance within tumors. The GEPIA database is a public resource for gene expression analysis, integrating large-scale data sets from tumor and normal tissues, with interactive tools for differential expression, survival analysis, correlation, and other functional analyses (61).

### MTT assay and calcein-AM/PI staining

Cells co-cultured with *E. faecalis* were further incubated for 48 h. For the MTT assay, 3-(4,5-dimethylthiazol-2-yl)−2,5-diphenyltetrazolium bromide (MTT; 2.5 mg/mL, 20 μL/well) was added in the dark and incubated for 4 h. The supernatant was then removed, and dimethyl sulfoxide (DMSO; 150 μL/well) was added. The mixture was shaken for 15 min, and absorbance was measured at OD_570_. For calcein-AM/PI staining, following co-culture, calcein-AM/PI solution (Beyotime, 250 μL/well) was added, and the cells were incubated at 37°C and 5% CO_2_ for 30 min. After washing with PBS, the cells were imaged using a fluorescence microscope. In the inhibitor assay, the TLR2/4 inhibitor sparstolonin B (SsnB; 5 mM) or the ROS scavenger NAC (5 mM) was applied to investigate the roles of the TLR signaling pathway and ROS in V583-induced cell proliferation. FSL-1 (GlpBio, 0.05 μM) was also employed as a reference control to evaluate its proliferative effects and functional interaction with V583.

### Immunofluorescence staining of Ki67 and EdU assay

Pancreatic cancer cells stimulated by V583 were further cultured for 48 h. The cells were fixed with 4% paraformaldehyde, permeabilized with 0.5% Triton X-100 for 10 min at room temperature, and washed three times with PBS. Endogenous peroxidase activity was blocked with serum, and the cells were incubated overnight at 4°C with anti-Ki67 primary antibody (Abclonal, 1:1000). After PBS washing, a Cy3-conjugated secondary antibody (Jackson ImmunoResearch, 1:200) was applied for 1 h, followed by counterstaining with DAPI. Fluorescence was then observed under a fluorescence microscope. At various time points, EdU working solution was added according to the manufacturer’s instructions (Beyotime, China), and the cells were incubated at 37°C for 3 h. Following fixation, permeabilization, and blocking, the Click reaction solution was added and incubated in the dark for 30 min. After washing, streptavidin-HRP working solution was added and incubated for 30 min. The cells were washed with PBS, and chromogenic solution was added, and the absorbance at OD_370_ was measured using a microplate reader.

### Cell scratch and transwell chamber assay

*E. faecalis*-stimulated cells were seeded into 24-well plates and cultured in medium supplemented with triple antibiotics. A uniform scratch wound was created by gently scraping the monolayer with a sterile pipette tip. The cells were then washed with PBS and cultured in medium containing 1% FBS and triple antibiotics. Wound closure was monitored by capturing images at 0, 24, and 48 h using an inverted microscope. For the Transwell assay, Matrigel-coated chambers were used to assess cell migration. Cells were seeded into the upper chamber, while the lower chamber was filled with complete medium. After incubation at 37 °C for 48 h, the migrated cells were fixed with 4% paraformaldehyde and stained with 1% crystal violet. Images were captured using an inverted microscope. Finally, the crystal violet was dissolved in 33% glacial acetic acid, and the absorbance at 570 nm was measured using a microplate reader.

### Western blotting

Cells were lysed using RIPA buffer (Beyotime, China), and protein concentrations were determined using the BCA method. Equal amounts of protein samples were loaded onto SDS-PAGE for separation and then transferred onto PVDF membranes (Millipore). The membranes were blocked and incubated overnight at 4°C with specific primary antibodies against EGFR, p-EGFR, mTOR, p-mTOR, cleaved caspase 3, caspase 9, PCNA, cyclin D1, E-cadherin, N-cadherin, AKT, and p-AKT. After washing with TBST, the membranes were incubated with secondary antibodies (goat anti-rabbit or goat anti-mouse, Proteintech) at room temperature for 2 h. Protein bands were visualized using chemiluminescent imaging.

### Intracellular ROS detection

Cells were seeded into culture plates, after attachment, divided into control and V583 infection (MOI = 10) groups. Cells were co-cultured with bacteria for 4 h, washed to remove extracellular bacteria, and then cultured for 48 h in medium containing triple antibiotics. Next, 2 μM DCFH-DA (Aladdin, Shanghai, China) was added to each well, followed by 30 min of dark incubation to allow probe uptake and conversion by intracellular esterases to DCFH, which is oxidized by ROS to form fluorescent DCF. After washing with PBS to remove unbound probe, fluorescence was measured using a fluorescence microscope. To assess the role of ROS in V583-induced effects, 5 mM NAC was added after co-cultivation, and after 48 h, cell viability and Western blot assays were conducted.

### Cytokine microarray and RNA-Seq

The Human XL Cytokine Array Kit (R&D, ARY022B) was employed to analyze multiple cytokines. Following a 4-h co-incubation of PANC1 cells with V583, the supernatants were collected and filtered through a 0.22-μm sterile filter to obtain the conditioned medium. According to the manufacturer’s instructions, chemiluminescence images were captured, and the changes in cytokine content in the conditioned medium were analyzed.

After V583 stimulation, PANC1 cells were harvested 48 h later, washed with PBS, digested with trypsin, and centrifuged at 1,000 × *g* for 5 min. The supernatant was discarded, and Trizol was added to homogenize the cells. The mixture was then aliquoted into RNase-free cryotubes, rapidly frozen in liquid nitrogen, and sent to Majorbio (Shanghai, China) for sequencing on the Illumina NovaSeq 6000 platform.

### Animal study

To evaluate the *in vivo* effects of V583 on pancreatic cancer growth, a PANC02 tumor-bearing mouse model was established. C57BL/6 mice were obtained from the Experimental Animal Center of Shaanxi Normal University. Each mouse was subcutaneously injected with 4 × 10⁶ PANC02 cells. When tumor volumes reached approximately 100 mm³, V583 (8 × 10⁸ CFU per mouse) was administered via intratumoral injection five times at three-day intervals; the control group received an equal volume of BHI medium. Tumor growth was monitored throughout the experimental period, and tumor tissues were collected at day 60. Paraffin-embedded sections were prepared, and hematoxylin and eosin (H&E) staining as well as immunohistochemical staining were performed to assess tissue architecture, cellular morphology, and the expression levels of Ki67 and EGFR.

### Statistical analysis

Data are expressed as mean ± standard deviation (SD). Statistical comparisons between groups were conducted using one-way ANOVA. A *P*-value less than 0.05 was considered statistically significant.

## Data Availability

The data sets used in this work are available from the corresponding author upon reasonable request. Raw sequencing data have been deposited in the in the NCBI Sequence Read Archive under accession number PRJNA1433153.
